# Bladder-draining lymph nodes support germinal center B cell responses during urinary tract infection in mice

**DOI:** 10.1128/iai.00317-23

**Published:** 2023-10-26

**Authors:** Sophia Hawas, Dimitrios Vagenas, Ashraful Haque, Makrina Totsika

**Affiliations:** 1 Centre for Immunology and Infection Control, School of Biomedical Sciences, Faculty of Health, Queensland University of Technology, Brisbane, Queensland, Australia; 2 Research Methods Group, School of Public Health and Social Work, Faculty of Health, Queensland University of Technology, Brisbane, Queensland, Australia; 3 Department of Microbiology and Immunology, University of Melbourne, The Peter Doherty Institute for Infection and Immunity, Parkville, Victoria, Australia; Universite de Geneve, Geneva, Switzerland

**Keywords:** UPEC, UTI, adaptive immunity, B-cell responses, germinal center, bladder, lymph nodes, multidrug resistance, flow cytometry, urine

## Abstract

Bacterial urinary tract infections (UTIs) are both common and exhibit high recurrence rates in women. UTI healthcare costs are increasing due to the rise of multidrug-resistant (MDR) bacteria, necessitating alternative approaches for infection control. Here, we directly observed host adaptive immune responses in acute UTI. We employed a mouse model in which wild-type C57BL/6J mice were transurethrally inoculated with a clinically relevant MDR UTI strain of uropathogenic *Escherichia coli* (UPEC). Firstly, we noted that *rag1^−/−^* C57BL/6J mice harbored larger bacterial burdens than wild-type counterparts, consistent with a role for adaptive immunity in UTI control. Consistent with this, UTI triggered in the bladders of wild-type mice early increases of myeloid cells, including CD11c^hi^ conventional dendritic cells, suggesting possible involvement of these professional antigen-presenting cells. Importantly, germinal center B cell responses developed by 4 weeks post-infection in bladder-draining lymph nodes of wild-type mice and, although modest in magnitude and transient in nature, could not be boosted with a second UTI. Thus, our data reveal for the first time in a mouse model that UPEC UTI induces local B cell immune responses in bladder-draining lymph nodes, which could potentially serve to control infection.

## INTRODUCTION

Urinary tract infections (UTIs) are one of the most common bacterial infections globally and contribute around US$ 6 billion to the annual global healthcare burden (1, 2). UTIs have a high rate of recurrence; over 30% of women with a primary infection will have a subsequent recurrent episode (1). Most UTIs are caused by uropathogenic *Escherichia coli* (UPEC) which are becoming increasingly multidrug-resistant (MDR) bacteria (3). Drug resistance is a major problem in the treatment of UTIs, and a higher proportion of infections are now becoming recurrent, leading to complicated UTIs (4). EC958, a representative multi-drug resistant strain of the most dominant clinical lineage circulating worldwide, has been shown to persist in the intestinal and urinary tracts of mice and humans (3, 5 – 8). As such, investigating adaptive immune responses to EC958 would be beneficial for therapeutic development and for understanding its mechanisms of persistence. Currently, there is some evidence of adaptive immunity during UTI and the role it plays in affecting infection outcomes. Antigen-presenting cells such as dendritic cells (DCs) are present during the inflammatory innate response, recruited by cytokines released by neutrophils and macrophages (9 – 11). Given their presence during innate immunity, it has been theorized that dendritic cells are responsible for ushering in the adaptive immune response and recruiting T and B cells to the bladder via antigen presentation and trafficking to local lymph nodes (9, 10, 12, 13). Previous studies in mouse knockout models of key immune cells (dendritic, T, and B cells) have shown that adaptive immunity is important for multiple UTI outcomes, such as susceptibility to colonization, bacterial clearance, and protection from subsequent infection (10, 14, 15). In a mouse model investigating the role of macrophages in UTI, Mora-Bau et al. found evidence of an adaptive immune response through recruited T and B cells to the bladder, which, however, did not offer sterilizing immunity to infected mice (10). A key cytokine in innate UTI immunity is interleukin (IL)-17, the majority of which is generated not only by bladder-resident γδ T cells (14) but also by innate lymphoid cells in humans (16). Deficiency in this cytokine predisposes mice to bacterial persistence in the kidneys (14). In a γδ T cell-deficient mouse strain, mice had significantly higher bladder bacterial burdens compared to immunocompetent mice (15). A major component of adaptive immunity is the humoral (B cell mediated) response, which is responsible for antibody production and immunological memory.

B cells are responsible for a wide variety of effector functions in humoral immunity, and naïve B cells require stimulation from an antigen, delivered to them by antigen-presenting cells (17). Naïve B cells in lymphatic tissue undergo maturation when exposed to an antigen, in a site within the lymph node known as the germinal center (GC). Here, they mature into activated GC B cells which are the progenitor cell line for B cell subsets such as plasma and memory B cells (17), whose primary roles are to regulate antibody production. These cells are known to express markers such as Fas (CD95) and GL7, which are specific to activated B cells undergoing maturation within the dark zone of the germinal center (17 – 19). In UTI vaccination studies, experimental vaccines have been shown to elicit protective effects and stimulate antibody production in animal model infections (20 – 22), implying that antibody production during UTI is correlated with better infection outcomes. In a small cohort of human patients, few people with lower UTI had detectable antibody-secreting cells in their urine during the first few weeks of infection (20). In a monkey model of cystitis, anti-*E*. *coli* antibodies in urine peaked by 5 weeks post inoculation (wpi) (22), indicating that while the B cell response in UTI may not be as robust as in other infections, the contribution of these cells nonetheless is important for infection outcomes. Additionally, the presence of antibodies in serum and urine during acute UTI in both mouse and monkey models is associated with bacterial clearance and reduced bacterial numbers in urine (21, 22). These antibody responses arise from GC B cells, making them an important cellular target for UTI responses. To date, the contribution and dynamics of these GC B cells have not been explored in the mouse UTI model.

Here, we report, for the first time, the induction of B cell responses by observing GC B cells during acute UTI in mice via flow cytometry, building on previous studies of innate immunity and UTI vaccinology. We first confirmed that adaptive immunity is important for infection outcomes and for controlling bacterial burden. We observed that GC B cells are generated in bladder draining lymph nodes during acute UTI but are transient, and their population does not increase by secondary infection administered a week following the initial infection. Together, our study shows for the first time that humoral immunity is induced locally in bladder-draining lymph nodes in UTI, which may have implications for UTI vaccine and drug development.

## RESULTS

### Adaptive immunity contributes to acute UTI control

**Fig 1:**
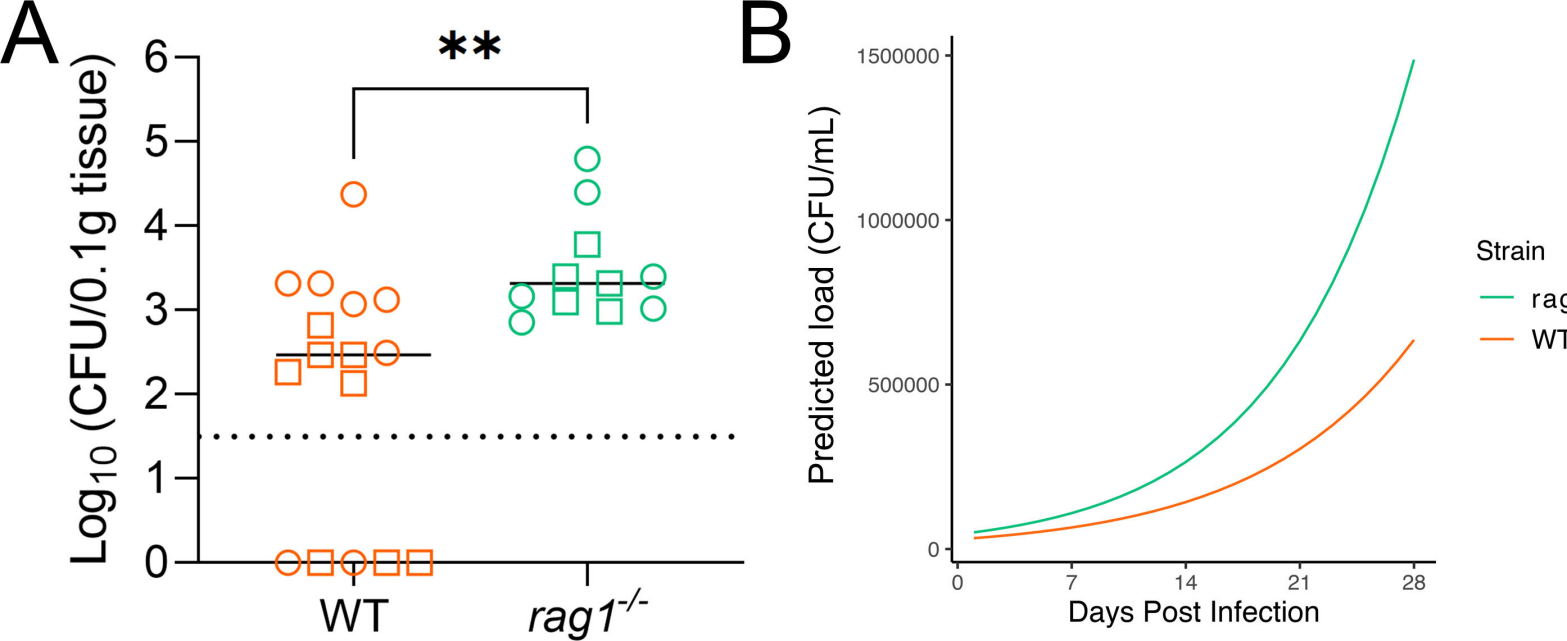
Impaired clearance of EC958 from the *rag1^−/−^* mouse bladder. (**A**) Scatter plot of C57BL/6 WT (*n* = 16) and *rag1^−/−^* (*n* = 11) mouse bladder EC958 colonization data (CFU/0.1 g bladder tissue) at 4 wpi from two independent experiments. Lines represent group medians, dotted line represents limit of detection, and ** *P* < 0.01 (Mann-Whitney test). (**B**) Longitudinal urinalysis showing the combined output of predicted colonization chance and bacterial load in urine (CFU/mL) during UTI in WT (*n* = 26) and *rag1^−/−^* mice (*n* = 11). Lines represent the overall predictions of a Zero Inflated Negative Binomial Mixed Model on urine data collected from three independent experiments.

To investigate the overall contribution of adaptive immunity to UPEC control during acute UTI, we compared bladder and urine bacterial loads in wild-type (WT) (C57BL/6) and *rag1^−/−^* mice (lacking mature T and B cells) experimentally inoculated with the reference MDR ST131 UPEC strain EC958 (3) in the bladder. At 4 weeks post inoculation (wpi), *rag1^−/−^* mice had 1-log higher median bacterial loads in the bladder compared to wild-type mice (*P* = 0.0017, unpaired non-parametric Mann-Whitney test) (Fig. 1A). Colony-forming units (CFU) in urine were collected over the course of the infection and analyzed using a series of statistical models (including mixed, additive, and zero inflated models) for parsimony, with the best fit being a Zero Inflated Negative Binomial Mixed Model (ZINBMM). Urinalysis using ZINBMM also confirmed overall higher susceptibility for *rag1^−/−^* mice, where modeling of urine data collected over time from three independent experiments predicted a higher susceptibility to initial colonization for *rag1^−/−^* mice. However, once colonized, either mouse strain is predicted to have the same urinary bacterial burden over time. Combined, this results to an overall higher bacterial burden predicted for the *rag1^−/−^* mouse group compared to WT mice (Fig. 1B). Taken together, *rag1^−/−^* mice are more susceptible to acute UTI, demonstrating a role for adaptive immunity in infection control.

### Dendritic and other innate immune cells are recruited to the bladder during acute infection

**Fig 2:**
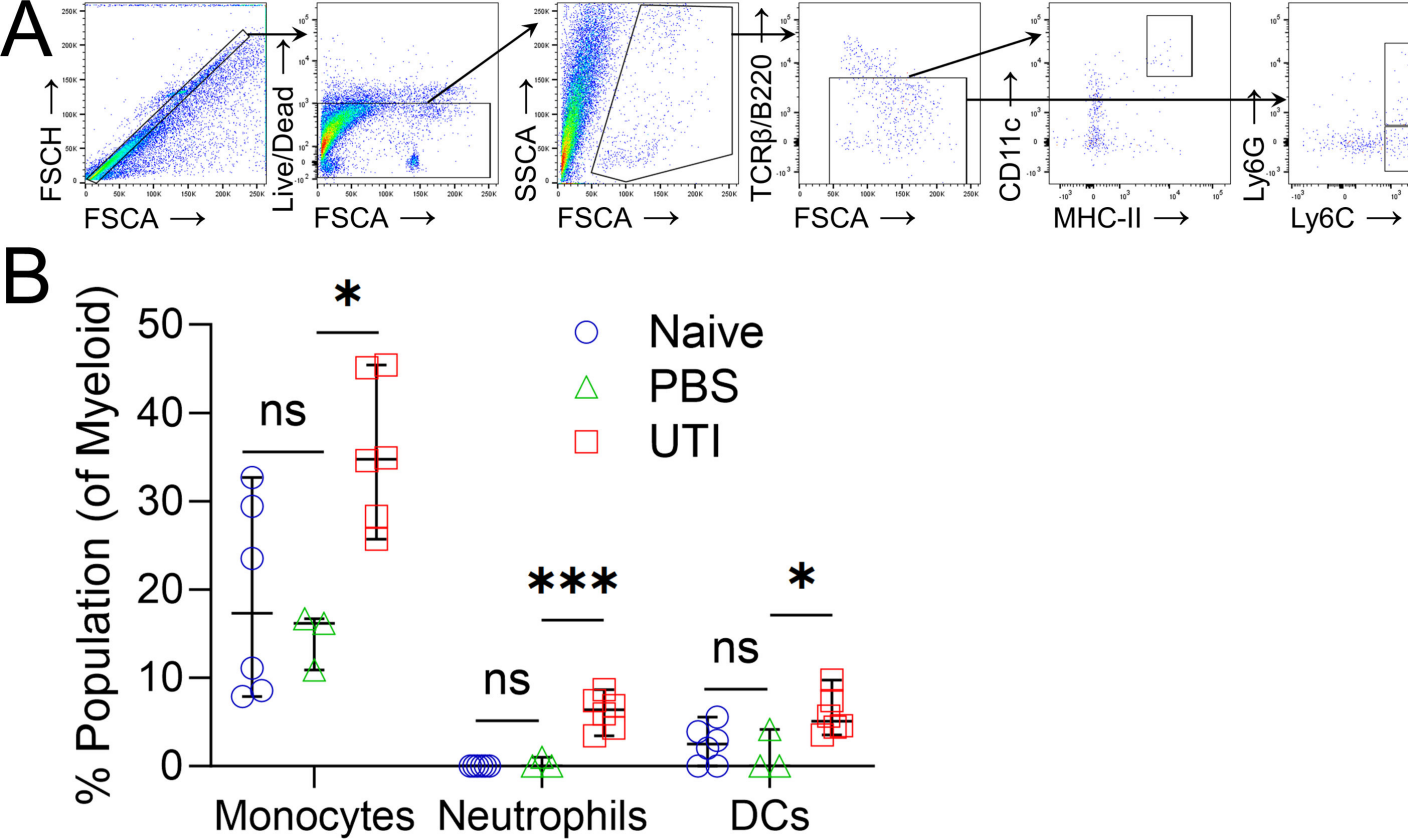
Antigen presenting cells (APCs) (monocytes, neutrophils, and dendritic cells) are recruited to the C57BL/6 mouse bladder 1 day post inoculation with EC958. (**A**) Fluorescence-activated cell sorting (FACS) gating strategy for myeloid cells, after gating on single live lymphocytes, B220^−^ TCRβ^−^ populations were characterized as myeloid cells within bladders. Within the myeloid population, markers MHC-II, CD11c, Ly6G, and Ly6C further characterised cell subsets, such as monocytes (Ly6C^hi^ Ly6G^lo^), neutrophils (Ly6C^hi^ Ly6G^hi^), and dendritic cells (DCs) (MHC-II^+^ CD11c^+^). (**B**) Myeloid populations present in bladders of mice that were naive, transurethrally catheterized, and inoculated with EC958 or mock-catheterized with saline phosphate-buffered saline (PBS). Group differences detected by Kruskal-Wallis test with Dunn’s post hoc test (**B**); bars represent median ±95% confidence Interval (CI); ns, not significant; * *P* < 0.05; *** *P* < 0.001.

A robust innate immune response has been previously demonstrated in several UTI mouse studies (9, 11, 14, 21, 23 – 29). We confirmed that this also occurs with EC958, where after 24 hours of inoculation, we observed increased infiltration of monocytes (*P* = 0.026), neutrophils (0.002), and dendritic cells (0.026) into the bladder of C57BL/6 WT mice in the UTI group compared to naïve controls (Fig. 2B). Bladder and kidney bacterial loads at this timepoint were also assessed and found to be comparable to previous studies using the same mouse-UPEC strain combination (Fig. S1A) (3).

### B cell activation occurs locally and transiently during acute UTI

**Fig 3:**
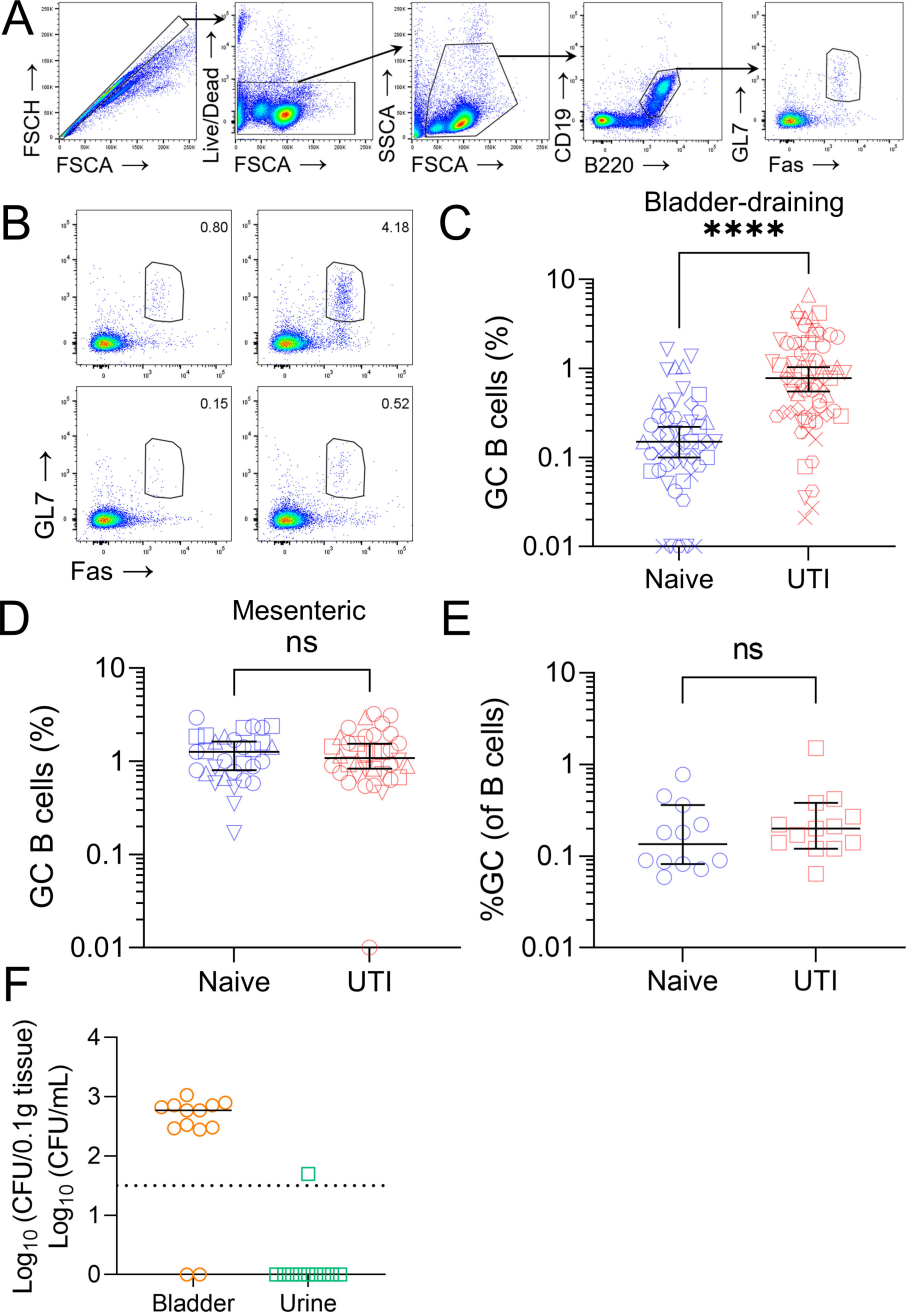
B cell activation occurs in bladder-draining lymph nodes at 4 weeks following acute UTI in C57BL/6 mice. (**A**) FACS gating strategy for GC B cells and (B) representative plots of the GC B cell gate in bladder-draining lymph nodes from two mice per group (UTI top, naïve bottom), percent GC B cells (of total B cells detected) in (C) bladder-draining lymph nodes at 4 wpi (UTI, *n* = 67; naïve, *n* = 52), (**D**) mesenteric lymph nodes at 4 wpi (UTI, *n* = 36; naïve, *n* = 31), (**E**) bladder-draining lymph nodes at 7 wpi (UTI, *n* = 13; naïve, *n* = 12), (**F**) bacterial load in UTI mouse bladders (CFU/0.1 g tissue) and urine (CFU/mL) at 7 wpi (*n* = 13). Lines in panels B–E represent group medians ± 95% CI, line in panel F represents median, and dotted lines represent LOD. Group differences detected by Mann-Whitney test; ns, not significant; ****P < 0.0001.

Despite the role of adaptive immunity in UTI, bladder draining lymph node B cell subsets have not been previously reported in the UTI mouse model. Given the extensive investigation into the innate immune response in this model, specifically, the recruitment of dendritic cells to the bladder, we hypothesized that B cell responses are triggered in bladder-draining lymph nodes in UTI. We assessed bladder-draining lymph nodes of both naïve and inoculated mice at 4 weeks specifically to observe expected B cell responses at their peak via flow cytometry (30). After gating on single live lymphocytes, B220^+^ CD19^+^ populations were characterized as B cells within lymph nodes and were found to be no different in proportion between naïve and UTI mice (Fig. S2A). Within the B cell population, we used the markers IgD, CD138, Fas (CD95), and GL7 to further define B cell subsets, such as GC B cells (Fas^+^ GL7^+^) and plasmablasts (IgD^lo^ CD138^+^) (Fig. 3A; Fig. S2C). Additionally, we confirmed the presence of germinal center structures in mesenteric lymph nodes from UTI group mice by confocal microscopy (Fig. S2D). We attempted to correlate bladder bacterial load and GC B cell percentage in UTI mice using simple linear regression; however, we did not observe any correlation between these outcomes (Fig. S2B).

At 4 weeks post UPEC inoculation, we observed an increase in GC B cells in bladder-draining lymph nodes of C57BL/6 UTI mice (Fig. 3C). While the proportion of GC B cells was relatively low (median, 0.78%), this cell population was statistically larger than in the naïve mouse group (median, 0.15%) (*P* < 0.0001), providing supporting evidence for the induction of a local humoral response in UTI. However, we observed no differences in GC B cells in distal mesenteric lymph nodes (Fig. 3D) or increases in plasmablast populations between groups (Fig. S2C). This local response was also transient (Fig. 3E), despite detectable CFU remaining present in the bladders of infected mice at 7 wpi (Fig. 3F).

### The GC B cell population in bladder-draining lymph nodes of UTI mice remains the same following re-infection

**Fig 4:**
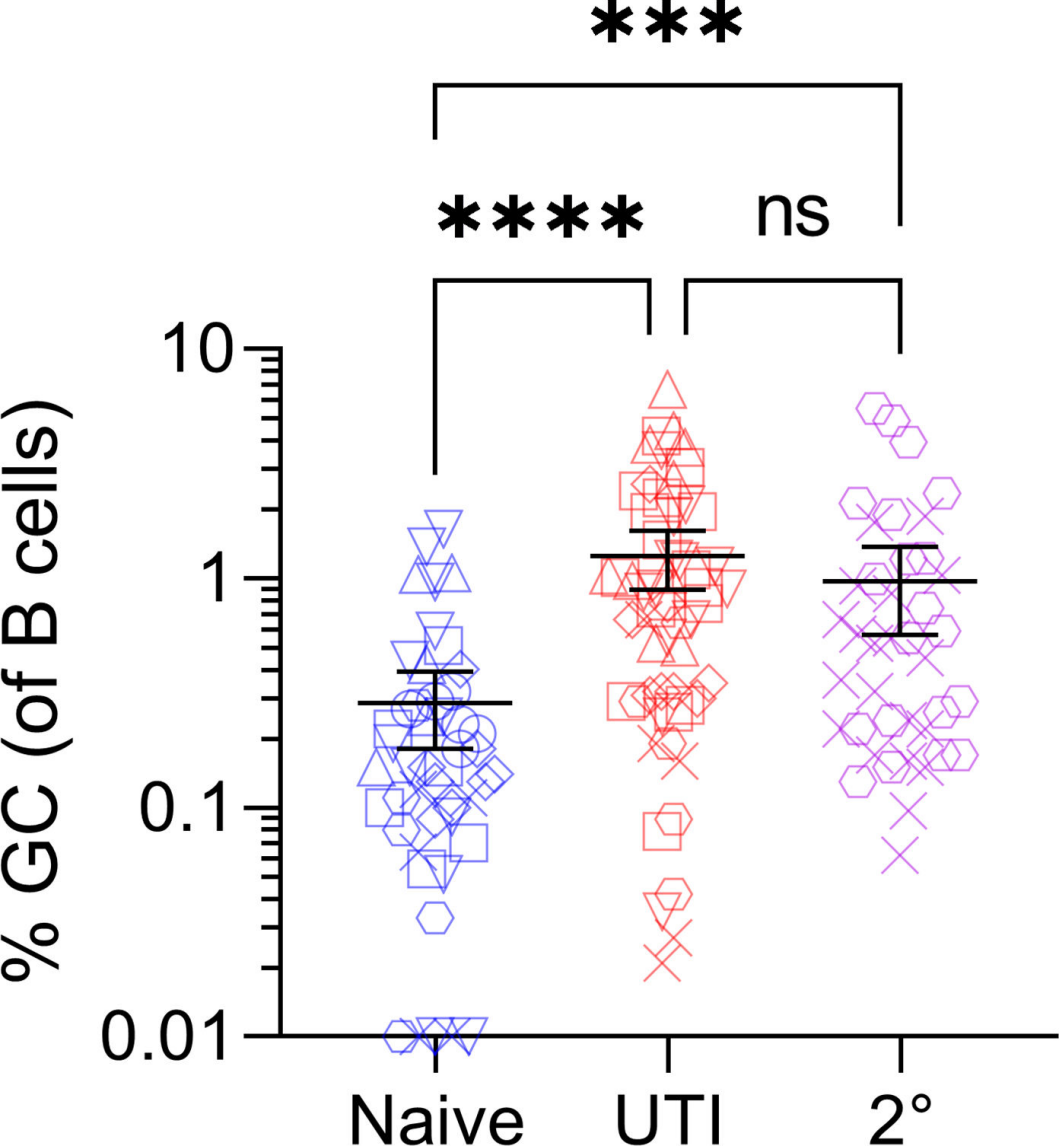
Secondary EC958 inoculation after the first week of acute UTI does not increase the proportion of GC B cells present in local lymph nodes in C57BL/6 mice. Percentage of GC B cells of the total number of B cells detected at 4 weeks in bladder-draining lymph nodes of C57BL/6 naïve mice (*n* = 52), mice receiving a single dose of UTI inoculum (UTI: *n* = 67), or a secondary inoculation (2°) 1 week after the primary (*n* = 40). Group differences detected by Kruskal-Wallis test with Dunn’s post hoc test; bars represent median ± 95% CI; ns, not significant; *** *P* < 0.001; **** *P* < 0.0001.

Given the transient nature of the B cell response, we hypothesized that it could be boosted by administering a secondary inoculation of UPEC 1 week following the initial inoculation, due to increased antigen availability and uptake. At 4 weeks, C57BL/6 mice that had experienced a single UTI episode or were re-infected after 1 week with the same UPEC strain had the same proportion of GC B cells in their bladder-draining lymph nodes (Fig. 4) and similar urinary organ colonization despite receiving a second inoculation (Fig. S3A). Both infected mouse groups had statistically increased GC B cell populations compared to naïve mice in bladder draining lymph nodes (*P* = 0.0002). Re-infected mice had statistically lower GC B cell populations in their mesenteric lymph nodes compared to both naïve and single-infection (UTI) mice [Fig. S3B; *P* = 0.0034 (naïve), 0.0022 (UTI)].

## DISCUSSION

In this study, we provide evidence consistent with the hypothesis that local humoral immune responses are generated during UTI in mice. Firstly, using *rag1^−/−^* mice, we conclude that adaptive immunity likely contributes to infection control. Both *rag1^−/−^* and wild-type mice have intact innate immune responses; therefore, differences in bladder colonization are most likely a difference in adaptive immune responses. Given that these responses are classically generated later in infection, we can speculate that this early difference may be caused by bladder-resident T and B cells, which would be activated much faster than systemic adaptive immunity. Regardless, this suggests a role for adaptive immunity as a whole in UTI, which is demonstrated here in terms of bladder colonization differences at later timepoints (Fig. 1A), as no difference is seen at early timepoints (~18 hours post inoculation) (16). Furthermore, it has previously been demonstrated that *rag1^−/−^* mice are susceptible to chronic bacteriuria (31), which is in line with our statistical modeling of urinary bacterial burden (Fig. 1B). Importantly, we show for the first time that GC B cell responses are generated in bladder-draining lymph nodes during acute UTI. However, these responses appear to be relatively modest and transient in nature and were not boosted upon bladder re-infection. Given previous reports of B cells and/or bacteria-specific antibody generation during UTI in both mice and humans (32 – 35), our data suggest that at least some of the upstream T and B cell immune responses that lead to antibody production likely occur in local draining lymph nodes.

Transient GC B cell responses are not unusual in other infection settings, since they subside as the prevalence of antigen decreases (17). However, we observed that despite the return of GC B cell proportions to baseline by 7 wpi, detectable bacteria still remained in bladders. This implies that the local immune response is insufficient to eradicate bacteria, a claim supported by a study conducted by Zychlinsky et al. that focused on sex differences between male and female UTI responses (36). Here, they suggest that an adaptive response mounted to a challenge infection is capable of reducing bacterial burden but not fully eradicating it (36). UPEC employ a variety of mechanisms to avoid clearance, including the formation of intracellular bacterial communities and quiescent intracellular reservoirs (37, 38) and blocking of TLR4 and cytokine signaling (23). Hence, we speculate that bacterial colonization strategies in this model are able to survive and perhaps even subvert the host humoral immune response.

The magnitude of the GC B cell response varied substantially among individual mice. Reasons for this variability are unclear but may have arisen from differences in innate immune response, which has previously been shown to affect infection outcomes (24, 39). High and transient levels of tumor necrosis factor alpha (TNFα), IL-6, and IL-8 are associated with bacterial clearance, whereas sustained cytokine levels for prolonged periods of time are associated with tissue damage in the bladder (23, 24, 39). Pre-existing antibody titers against the infecting strain also affect the rate of bacterial clearance (40), suggesting that pre-existing antibodies to commensal organisms in “specific pathogen-free” conditions might have influenced outcomes, although how this might induce variation between co-housed mice is unclear. Interestingly, secondary inoculation of mice did not lead to increased clearance rates compared to mice infected once, suggesting either that there was insufficient time for titers to develop or that the GC B cell response overall is not strong enough to produce meaningful antibody titers against the infecting UPEC strain EC958. The variation in responses could also be due to individual differences between mice, a common effect observed even in genetically identical mice (41).

A limitation of this study is that we were unable to conclude the individual contribution of each bladder-draining lymph node to the GC response, i.e., whether a specific draining lymph node generates more GC B cells than the others. In order to assess this, a more sensitive screening method could be employed, or the B cell population could be enriched to remove other cell types and improve accuracy (42). Similarly, we did not deplete B cells to identify their exact role during infection but rather focused on boosting the already present response in order to observe any potential enhancement as a result of this. A recently published article by Rousseau et al. assessed bladder bacterial burden after 4 weeks post inoculation in a B cell-deficient mouse background (μMT) and found that there was no difference in burden between B cell-deficient mice and the wild-type strain (43). While this does indeed suggest that B cells are dispensable for bacterial clearance, combined with our data, it also suggests that B cells are unlikely to contribute to bacterial clearance as a result of their weak response during acute UTI. We also observed that overall B cell (B220^+^ CD19^+^) proportions remained the same between naïve and UTI mice (Fig. S2A). While differences in GC B cells were small between naïve and UTI mice (median, 0.15% vs 0.78%; Fig. 3C), this represents a fivefold increase, and given the robustness and power of our data sets (*n* = 57 naïve, 67 UTI, over eight experimental repeats), we are confident that this reflects the cells present in bladder-draining lymph nodes. Whether the magnitude of such increase is biologically relevant for UTI remains to be determined.

On that note, the GC B cell response that we observed in UTI mouse bladder-draining lymph nodes is anecdotally weak compared to other infection models used in our groups (e.g., blood-stage *Plasmodium* spp. infection) (44), leading us to speculate that specific aspects of the bladder may contribute to this difference. For instance, the architecture of the bladder is structured in a way that is difficult for molecules and cells to enter or leave (45, 46). During cystitis, the most important anatomical structures are the superficial bladder cells that come into contact with UPEC and the contents of the bladder lumen, which are held together with tight junctions. These cells are covered with plaques called uroplakins which add additional protection against toxic compounds in urine and pathogens (46, 47). Insufficient antigen uptake from the bladder to nearby lymph nodes would also affect antigen presentation and subsequent activation of adaptive immune cells. In aged mice, it has been reported that tertiary lymph structures form as a result of chronic age-associated inflammation, involving the secretion of IgA from plasma B cells found in germinal centers of these structures (48 – 51). Additionally, an increased proportion of activated B cells was observed in female patients with interstitial cystitis, a condition characterized by consistent bladder inflammation (52). Taken together, this suggests that increased inflammation encourages local B cell activation and antibody secretion. Likewise, increased bladder damage has been reported to lead to a stronger immune response (39, 53), and we reasoned that administering a second UPEC inoculation, similarly performed in previous studies (21, 54, 55), might enhance antigen uptake and presentation, simulating the administration of an adjuvant directly to the site of infection. We reasoned that an additional inoculation after 1 week would be akin to receiving a dose of monophosphoryl lipid A, a modified component of Gram-negative lipopolysaccharide (and thus present on EC958), which is a commercially available adjuvant and has been used previously in UTI vaccine studies (56). The modest GC B cell responses could theoretically also be the result of adaptive immune suppression via cytokines, which is a widely cited reason for a lack of a strong adaptive response in the bladder (12, 57 – 60). Early during the innate immune response to UPEC, IL-10 is secreted by mast cells to dampen inflammation and prevent bladder damage (57, 61). This in turn decreases DC activation and CD4^+^ T cell and GC B cell responses in draining lymph nodes. Hence, we speculate that one possible method for boosting antibody production during UTI might be to transiently block IL-10 signaling, as it has been shown that IL-10 knockout mice can more efficiently clear UPEC from their bladders in early infection and generate an increased UPEC-specific antibody titer (57). We also saw the modest GC B cell response reflected by the spatial organization of GCs within lymph tissue (Fig. S2D). We observed evidence of PNA^+^ cells (indicative of GC B cells) in mesenteric lymph nodes of UTI mice, but we were unable to observe clear evidence of infection-induced punctate GC structures in the bladder-draining lymph nodes, likely due to technical challenges associated with the small and delicate nature of these tissues. Although our flow cytometry analysis clearly demonstrates an increase in the abundance of GC B cells in the draining lymph nodes after acute UTI, it appears that the scarcity of these cells makes GCs technically challenging to locate via microscopy.

Recently, advances have been made in understanding roles played by T cells during UTI (53, 54). A population of microbiota-dependent memory CD4^+^ T cells has been found to exist in bladder-draining lymph nodes, which respond to activation by DCs trafficking from the infected bladder (53). Interestingly, after secondary cystitis, there is preferential activation of Th2 cells within the bladder-draining lymph nodes by DCs, skewing the immune response to that of tissue repair and immune tolerance (53), which we also speculate was the reason behind observing a decrease in re-infected mouse mesenteric GC populations (Fig. S3B) . This process was found to be triggered by the exfoliation of bladder cells during infection, which upregulated a subset of these DCs (CD301b+ OX40L+). Skewing toward a Th2-dependent response was found to suppress bacterial clearance while amplifying the bladder’s tissue repair processes (53). Administration of a Th1-skewing adjuvant, however, was able to rectify this and again promote bacterial clearance (54). Taken together, these factors suggest that multiple host and pathogen factors contribute to the severity of infection and the type of immune response that occurs during UTI.

In conclusion, we have robustly demonstrated that a humoral response is generated locally during UTI, and while there is a spread in responses within mouse cohorts, this is consistent across all experimental repeats. We also observed that this response is relatively short-lived. Further research into the types of B cell subsets present and how the GC B cell response could be amplified by vaccination or immunotherapy would be of great benefit to the UTI field. Boosting GC B cell responses with a range of adjuvants and/or immune-modulatory treatments could serve to improve protective immunity to UTI, given the modest response observed here. Finally, shedding light on strategies for boosting adaptive immunity in UTI could pave the way to lowering the rate of recurrence for one of the most common bacterial infections in humans.

## MATERIALS AND METHODS

### Bacterial strain and culture conditions

UPEC strain EC958 was used in this study as a reference multidrug-resistant ST131 strain (62). To promote the expression of type 1 fimbriae (T1F), EC958 was routinely cultured from highly fimbriated stocks, which were incubated overnight statically at 37°C. Cultures were assessed for expression of T1F by yeast cell agglutination as previously described (63) and were fixed at the inoculation cell density and volume (64).

### Mouse UTI model

Six- to seven-week-old female C57BL/6 and 8–17-week-old *rag1^−/−^* mice were catheterized as previously described (65). Briefly, mice were anesthetized by isoflurane inhalation and catheterized with ~1–2 × 10^8^ CFU EC958 in 30 µL. The prepared inoculum was deposited directly into the mouse bladder using a sterile catheter followed by immediate removal (65). A separate cohort of strain- and age-matched mice was used as controls and was not catheterized with bacteria. For innate immunity experiments, an additional cohort of mice was mock catheterized with phosphate-buffered saline (PBS). For experiments involving multiple inoculations, secondary inoculation mice were catheterized as described above and then after 1 week were catheterized again with the same dose of bacteria. C57BL/6 mice were sourced from the ARC (Animal Resource Centre, Western Australia), and Mice were sacrificed at either 1 day, 4 weeks, or 7 weeks post inoculation, and the bladders, kidneys, and lymph nodes (lumbar aortic, medial iliac, mesenteric) were extracted.

### Flow cytometry

Bladders were incubated at 37°C for 1 hour in RPMI containing 1 mg/mL collagenase IV (Sigma, cat no. C1889-50MG, Darmstadt) and DNase I (Sigma, cat no. D4263-5VL, Darmstadt). Enzyme-treated bladders and lymph nodes were mechanically disrupted with a 100-µm cell strainer (Falcon, cat no. 352360, Corning, NY) and resuspended in fluorescence-activated cell sorting (FACS) buffer [1% fetal calf serum (FCS) in PBS]. Two hundred microliters of each sample were plated onto 96-well plate (Falcon, cat no. 353077, Corning, NY). Two draining lymph nodes were pooled per mouse. All samples were stained with Live/Dead Aqua (Invitrogen, L34965, Waltham, MA) and the respective antibody panels in Tables 1 and 2. Samples were acquired on the Cytoflex S (Beckman-Coulter, Brea, CA) and BD Fortessa (BD Biosciences, Franklin Lakes, NJ), and data were processed using FlowJo v10 (TreeStar, Franklin Lakes, NJ) software.

**TABLE 1:** B cell antibody panel used in this study

Fluorophore	Marker	Clone	Supplier	Cat no.
FITC	CD19	6D5	BioLegend	115506
eFluror660	GL7	GL-7	eBioscience	50-5902-82
Alexa Fluor 700	B220	6B2	BioLegend	103232
APC-Cy7	Surface IgD	26c.2a	BioLegend	405716
Brilliant Violet 421	Fas	Jo2	BioLegend	562633
Brilliant Violet 605	CD138	281–2	BioLegend	142516

**TABLE 2:** Myeloid antibody panel used in this study

Fluorophore	Marker	Clone	Supplier	Cat no.
FITC	Ly6C	HK1.4	BioLegend	128006
PerCp-Cy5.5	CD11b	M1/70	BioLegend	101228
APC	CD11c	N418	BioLegend	117310
Alexa Fluror 700	B220	RA3-6B2	BioLegend	103232
Alexa Fluror 700	TCR-β	H57-597	BioLegend	109224
APC-Cy7	Ly6G	1A8	BioLegend	127624
Pacific Blue	I/A-I/-E (MHC-II)	M5/114.15.2	BioLegend	107620

### Quantification of bacterial viable cell numbers

Bacterial loads were quantified over time in urine and at endpoint in infected mouse bladders. Urine samples were plated directly into a 96-well plate; bladders were homogenized in 50 µL of PBS using a Mini Beadbeater (BioSpec Products) before topping up to 1 mL with PBS and aliquoting 200 µL into the plate. Samples were serially diluted to 10^−4^ as previously described (65). Five microliters of each well for each sample were plated onto lysogeny broth (LB) agar (1% tryptone, 0.5% yeast extract, 0.5% salt, and 1.5% agar in Milli-Q water) in quadruplicate and then incubated overnight at 37°C. The following day, colonies were enumerated, and bacterial load was expressed as CFU/mL for urine or CFU/0.1 g of tissue for bladders.

### Statistical analysis

All statistical analysis was performed in GraphPad Prism Version 9 software (GraphPad Software) and R (66). Unpaired non-parametric Mann-Whitney or Kruskal-Wallis tests (with Dunn’s post hoc test) were used to test for statistically significant differences between group medians of uninfected (naïve) and UTI groups. Statistical significance was set at *P* < 0.05. Each unique symbol in Fig. 1, 3, and 4 denotes an experimental repeat.

#### Statistical modeling of longitudinal urinalysis data

For analysis of longitudinal urine bacterial load and susceptibility to colonization, a series of models were applied and tested in order to find the most appropriate model that fitted the nature of the data the best. Simple linear models were fitted with the log(CFU) as outcome and “Day “ and “Strain” as explanatory variables, where “Day” refers to the day post inoculation and “Strain” refers to mouse strain. The “lme” procedure from “nlme” package in R was then used to explore if a mixed model was needed, with “mouse” as a random effect, which would consider the potentially correlated nature of the data due to measurements taken on the same mouse over time. We also fitted a range of generalized additive models and generalized additive mixed models. These models are similar to the above, but instead of assuming a constant regression coefficient, they estimate a function(s) for the range of independent variables. Given the presence of a large number of zero values in the urine CFU data, zero inflated models were tested as well, as per Zuur et al. (67, 68). For these models, both a Poisson and a negative binomial distribution were fitted, with the latter potentially accommodating a variance larger than the mean (i.e., overdispersion), at the expense of estimating one more parameter compared to the Poisson distribution. From this analysis, the model which fitted the data best (as judged by the Akaike’s information Criterion) was a ZINBMM. R Packages used in this analysis included the following: “nlme,” “lme4,” “mgcv,” “glmmTMB,” “pscl,” “lmtest,” and “ggplot2” (69 – 75).

## Data Availability

The authors confirm that the data supporting the findings of this study are available within the article and its supplementary materials. Raw flow cytometry and bacterial quantification data supporting the findings of this study are available from the corresponding author (M.T.) on request.

## SUPPLEMENTAL MATERIAL

Supplemental file 1 iai.00317-23-s0001.docx Additional experimental details, supplemental Figures S1, S2, S3.

Supplemental file 2 iai.00317-23-s0002.xlsx Raw data table with urinalysis CFU values.

ASM does not own the copyrights to Supplemental Material that may be linked to, or accessed through, an article. The authors have granted ASM a non-exclusive, world-wide license to publish the Supplemental Material files. Please contact the corresponding author directly for reuse.
