# Supplementary material for: Bladder-draining lymph nodes support germinal center B cell responses during urinary tract infection in mice
Source: Infect Immun. 2023 Oct 26;91(11):e00317-23. doi: 10.1128/iai.00317-23 (PMC10652902; doi:10.1128/iai.00317-23)
Supplement: Supplemental file 1 — Additional experimental details, supplemental Figures S1, S2, S3. [file iai.00317-23-s0001.docx]

## **Supplementary Information for:** Bladder-draining lymph nodes support germinal centre B cell responses during urinary tract infection in mice.

Sophia Hawas^1^, Dimitros Vagenas^2^, Ashraful Haque^3^, Makrina Totsika^1#^

^1^Centre for Immunology and Infection Control, School of Biomedical Sciences, Faculty of Health, Queensland University of Technology, Brisbane, QLD, Australia.

^2^ Research Methods Group, School of Public Health and Social Work, Faculty of Health, Queensland University of Technology, Brisbane, QLD, Australia.

^3^Department of Microbiology and Immunology, University of Melbourne, The Peter Doherty Institute for Infection and Immunity, Parkville, Victoria, Australia.

#Address correspondence to Makrina Totsika, [makrina.totsika@qut.edu.au](mailto:makrina.totsika@qut.edu.au).

| **File** | **Name** | **Contents** |
| --- | --- | --- |
| 1 | Supplementary Information (this document) | Supplementary Methods, Supplementary Figures 1-3 |
| 2 | Supplementary File 2 Urine CFU long format | Raw input data used for ZINBMM analysis |

# Supplementary Information

## Supplementary Methods

### Fluorescence Microscopy

Lymph nodes were fixed with 2% paraformaldehyde for 48 h at 4°C in the dark followed by dehydration with 10% sucrose (Sigma) for 48 h at 4°C in the dark, and then 30% sucrose (Sigma) for 48 h at 4°C in the dark. Lymph nodes were snap-frozen in Tissue-Tek Optimal Cutting Temperature embedding medium (Sakura Finetek) on dry ice and stored at −80 °C. Lymph nodes were sectioned at 10 μm on polysine slides such that consecutive sectioning is avoided. Sections were allowed to dry overnight. The dried sections were rehydrated for 15–20 min before fixation in 4% paraformaldehyde for 15–20 min at RT in dark. Slides were washed 3 times for 5 min in wash buffer (0.01% Tween20 in PBS) before permeabilization with 0.1% Triton X-100 in wash buffer for 10–15 min. After washing, slides were incubated with Medical Background Sniper (Biocare) for 30 minutes. Slides were rinsed for 2 min and endogenous biotin was blocked using an Avidin/Biotin Blocking kit (Vector Laboratories), according to manufacturer’s protocol. Tissue sections were then stained with rat anti-mouse CD3-AF594 (1:200 dilution, clone: 1742, catalog no. 100240, Biolegend), rat anti-mouse B220-APC (1:200 dilution, clone: RA3-6B2, catalog no. 103212, Biolegend) and biotinylated peanut agglutinin (1:200 dilution, catalog no. B-1075-5 PNA; Vector Laboratories, Inc.) for 1–2 h at RT in the dark. Secondary antibody staining for PNA was performed using streptavidin–FITC (1:300 dilution, catalog no. 200-402-095, Thermo Fisher Scientific) for 1–2 h at RT in the dark. Tissue sections were incubated with DAPI for 10 min to counterstain nuclei, and slides were mounted in Dako Mounting Media (Agilent Technologies). Image acquisition was performed using a Zeiss 780-NLO confocal microscope at ×10 and x20 objective.


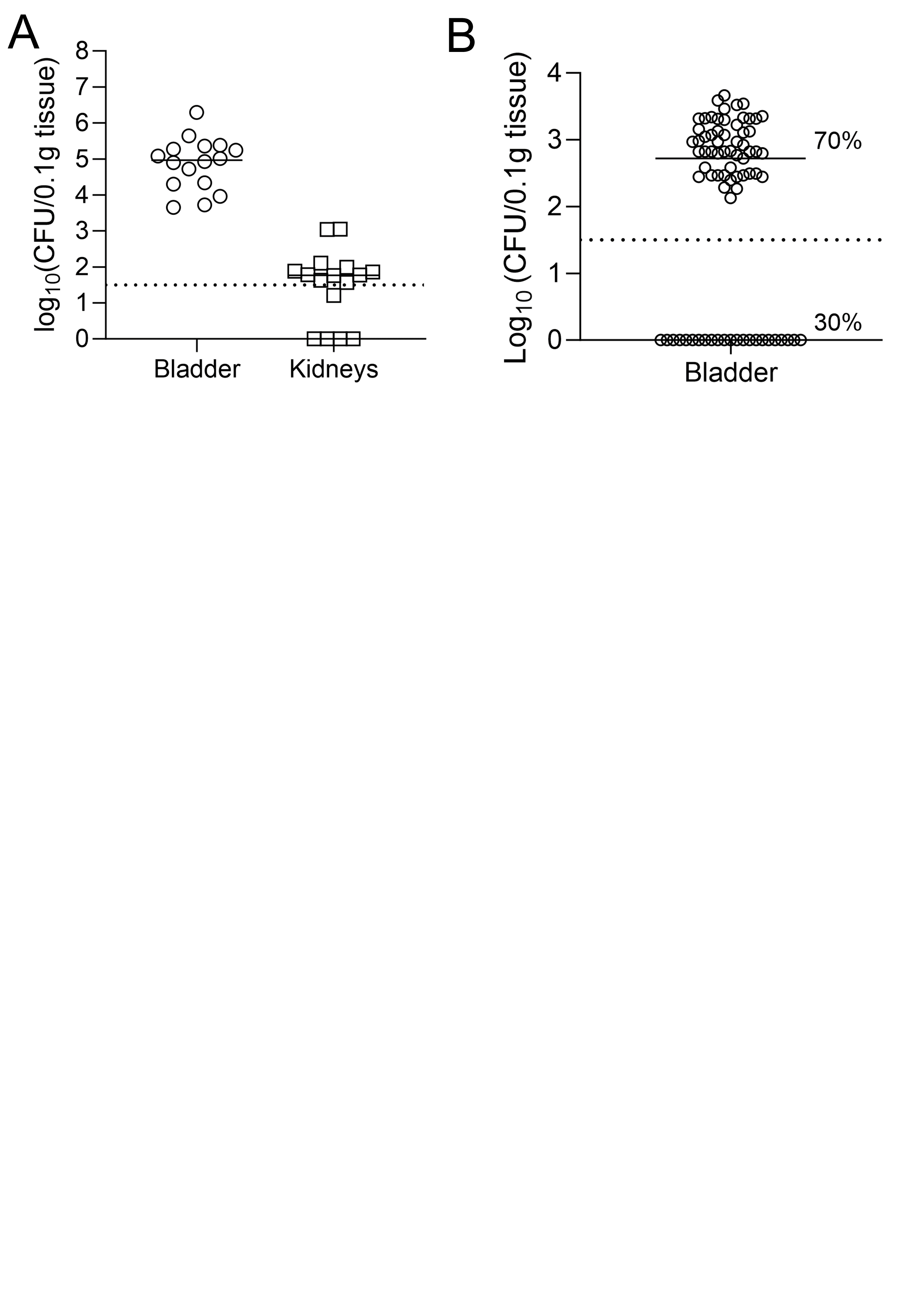


**Figure S1. Urinary organ bacterial load at 1 day and 4 weeks post inoculation.** A) Scatter plot of C57BL/6 (*n* = 16) bladder and kidney EC958 colonisation data (CFU/0.1g tissue) at 1 day post inoculation (dpi) from two independent experiments. B) Scatter plot of C57BL/6 (*n* = 67) mouse bladder EC958 colonisation data (CFU/0.1g bladder tissue) at 4 weeks post inoculation (wpi) from eight independent experiments, with percentages outlining the proportion of mice with recoverable bacteria at 4 weeks. Lines represent group medians, dotted line represents limit of detection (LOD).


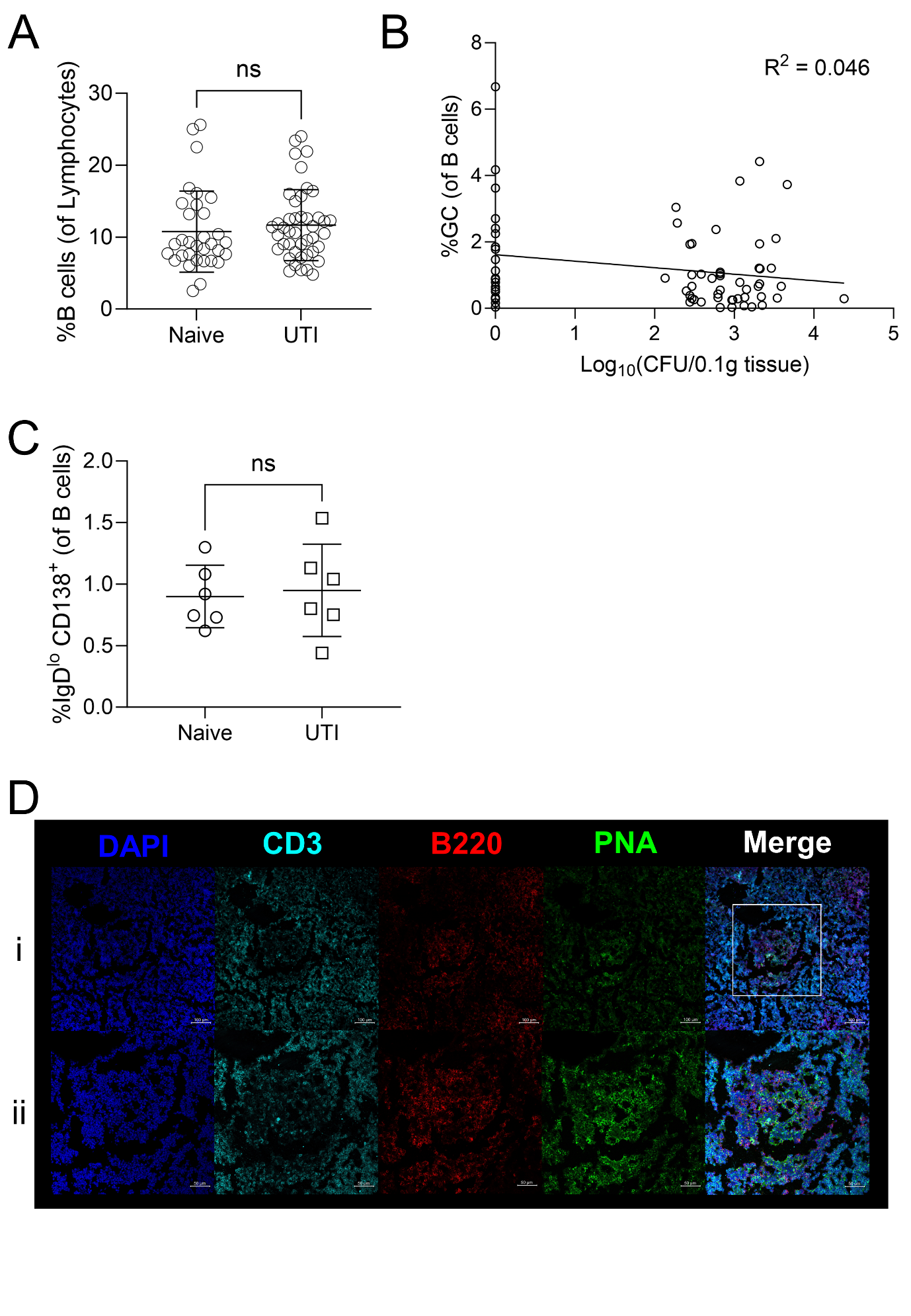


**Figure S2. Germinal centre (GC) B cells are localised within B cell follicles of bladder draining lymph nodes.** A) Total percentage of B cells in bladder draining lymph nodes of both naïve (*n* **=** 57) and UTI mice (*n* = 67), gating performed as shown in Figure 3, data from eight independent experiments, Mann-Whitney test, **ns** not significant. B) Simple linear regression of bladder EC958 load to GC B cell proportion in draining lymph nodes in UTI mice (*n* = 67), expressed as log_10_(CFU/0.1g tissue) vs. %GC (of B cells), data from eight independent experiments. C) Plasmablast populations in naïve (*n* = 6) and UTI (*n* = 6) mouse draining lymph nodes, gated as IgD^lo^ CD138^+^ cells, Mann-Whitney test, **ns** not significant. D) Immunofluorescence microscopy of a UTI mouse mesenteric lymph node section (10 μm) demonstrating a germinal centre structure. Tissue sections were stained with rat anti-mouse CD3-AF594 (1:200 dilution, clone: 1742, catalog no. 100240, Biolegend), rat anti-mouse B220-APC (1:200 dilution, clone: RA3-6B2, catalog no. 103212, Biolegend) and biotinylated peanut agglutinin (1:200 dilution, catalog no. B-1075-5 PNA; Vector Laboratories, Inc.) for 1–2 h at RT in the dark. Secondary antibody staining for PNA was performed using streptavidin–FITC (1:300 dilution, catalog no. 200-402-095, Thermo Fisher Scientific), tissue sections were also stained with DAPI to counterstain nuclei. Image acquisition was performed using a Zeiss 780-NLO confocal microscope at i) ×10 and ii) x20 objectives, scale bars = i) 100 μm, ii) 50 μm.

**
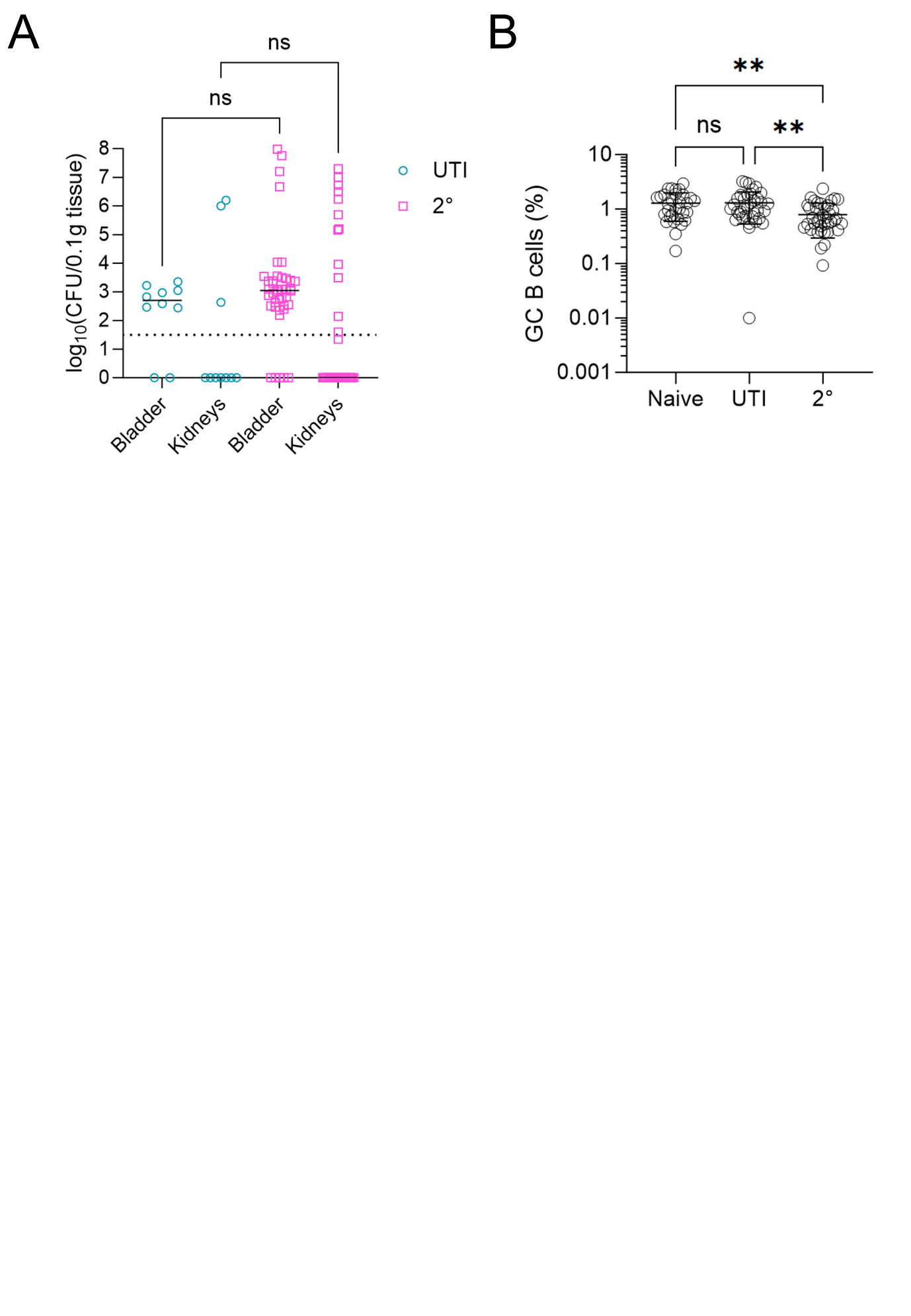
**

**Figure S3. Bladder and kidney bacterial load at 4 weeks post inoculation in acute UTI and in mice inoculated twice (2°).** A) Scatter plot of C57BL/6 bladder and kidney EC958 colonisation data (CFU/0.1g tissue) at 4 weeks post inoculation (wpi), UTI: *n* = 10, 2°: *n* = 40, two independent experiments*.* Group differences detected by Kruskal-Wallis test with Dunn’s correction, **ns** not significant, lines represent group medians, dotted line represents LOD. B) Germinal centre B cell populations present in mesenteric lymph nodes of mice that were naive, transurethrally catheterised and inoculated with EC958, or catheterised a second time one week after initial inoculation (2°). Group differences detected by Kruskal-Wallis test with Dunn’s correction, bars represent mean ± SD, **ns** not significant, ****** *p* <0.01.
